# AI in the Hot Seat: Head-to-Head Comparison of Large Language Models and Cardiologists in Emergency Scenarios

**DOI:** 10.3390/medsci14010033

**Published:** 2026-01-08

**Authors:** Vedat Cicek, Lili Zhao, Yalcin Tur, Ahmet Oz, Sahhan Kilic, Gorkem Durak, Faysal Saylik, Mert Ilker Hayiroglu, Tufan Cinar, Ulas Bagci

**Affiliations:** 1 Machine & Hybrid Intelligence Lab, Department of Radiology, Northwestern University, Chicago, IL 60611, USA; 2 Department of Preventive Medicine, Biostatistics and Informatics Division, Northwestern University, Chicago, IL 60611, USA; 3 Department of Computer Science, Stanford University, Stanford, CA 94305, USA; 4 Department of Cardiology, Istanbul Education and Research Hospital, Istanbul 34098, Turkey; drozahmet@gmail.com; 5 Corlu State Hospital, Tekirdag 59860, Turkey; 6 Department of Cardiology, Van Training and Research Hospital, Health Sciences University, Van 65100, Turkey; 7 Department of Cardiology, Dr. Siyami Ersek Cardiovascular and Thoracic Surgery Research and Training Hospital, Istanbul 34668, Turkey; 8 School of Medicine, University of Maryland, Baltimore, MD 21201, USA

**Keywords:** large language models, ChatGPT, myocardial infarction, catheterization laboratory

## Abstract

**Background:** The clinical applicability of large language models (LLMs) in high-stakes cardiac emergencies remains unexplored. This study evaluated how well advanced LLMs perform in managing complex catheterization laboratory (Cath lab) scenarios and compared their performance with that of interventional cardiologists. **Methods and Results:** A cross-sectional study was conducted from 20 June to 2 December 2024. Twelve challenging inferior myocardial infarction scenarios were presented to seven LLMs (ChatGPT, Gemini, LLAMA, Qwen, Bing, Claude, DeepSeek) and five early-career interventional cardiologists. Responses were standardized, anonymized, and evaluated by thirty experienced interventional cardiologists. Performance comparisons were analyzed using a linear mixed-effects model with correlation and reliability statistics. Physicians had an average reference score of 80.68 (95% CI 76.3–85.0). Among LLMs, ChatGPT ranked highest (87.4, 95% CI 82.5–92.3), followed by Claude (80.8, 95% CI 75.7–85.9) and DeepSeek (78.7, 95% CI 72.9–84.6). LLAMA (73.7), Qwen (66.2), and Bing (64.3) ranked lower, while Gemini scored the lowest (59.0). ChatGPT scored higher than the early-career physician comparator group (difference 6.69, 95% CI 0.00–13.37; *p* < 0.05), whereas Gemini, LLAMA, Qwen, and Bing performed significantly worse; Claude and DeepSeek showed no significant difference. **Conclusions:** This expanded assessment reveals significant variability in LLM performance. In this simulated setting, ChatGPT demonstrated performance comparable to that of early-career interventional cardiologists. These results suggest that LLMs could serve as supplementary decision-support tools in interventional cardiology under simulated conditions.

## 1. Introduction

The rapid development of artificial intelligence (AI) has significantly changed healthcare technology, with large language models (LLMs) emerging as a particularly promising innovation in medical diagnostics and clinical decision-making [1,2]. As LLMs quickly become part of public-facing platforms, their influence reaches beyond the clinical field affecting patient information, emergency triage, and even public trust in digital health systems [3]. While prior research has explored AI applications across various medical fields, the potential of advanced language models in high-stakes, time-sensitive medical settings remains largely unexplored. Cardiac catheterization labs (Cath lab) are a critical point where complex decisions, technological progress, and patient safety converge, making them an ideal environment to thoroughly evaluate the capabilities of emerging AI technologies [4]. Myocardial infarction (MI) management requires split-second decisions that can significantly impact patient outcomes in the Cath lab. Traditional medical training heavily depends on expert knowledge, clinical experience, and quick pattern recognition. Early-career cardiologists can find decision-making in the Cath lab challenging [4,5]. The introduction of LLMs offers a unique opportunity to improve and possibly strengthen this decision-making process. Still, the medical community has responded to these technologies with a mix of cautious optimism and genuine skepticism, recognizing both their transformative potential and the crucial need to ensure patient safety [6].From a public health perspective, assessing the reliability and safety of these tools is essential. Inappropriate or unsafe AI-generated medical advice can lead to systemic consequences, especially when models are freely accessible to the public [7].

Due to the complexity of medical language, LLMs have been adopted for various tasks in the medical field [8,9]. In cardiology, LLMs have demonstrated significant potential in identifying and characterizing cardiovascular disease groups, recognizing signs, symptoms, risk factors, comorbidities, and aiding clinical reasoning [10,11]. These models focus on low-stakes tasks, which can offer innovative solutions for patient education, clinical decision-making, enhancing research data collection, predicting diagnoses and cardiovascular disease outcomes from text, and reducing administrative burdens for healthcare providers. Recent studies have evaluated LLMs across several high-risk clinical domains, including automated ECG interpretation, emergency department triage, acute diagnostic reasoning, and time-sensitive cardiovascular decision-making outside the catheterization laboratory. These investigations have shown that LLMs can approximate clinician-level performance on selected cognitive tasks; however, most focus on diagnostic classification or text-based reasoning rather than procedural, operator-dependent decision-making. [12,13,14,15]. There has been no research examined the ideal use LLMs in high-stakes, especially in time-sensitive decision management scenarios, such as within the Cath lab. This study aims to show the potential of LLMs for managing emergencies and critical conditions and to compare their performance with early-career cardiologists in the Cath lab environment.

## 2. Methods

### 2.1. Overview of Large Language Models in Medical Practice

LLMs are deep neural network models with transformer-based architectures trained on large text datasets, capable of understanding context, generating human-like text, and handling complex language tasks [16]. The size of the model indicates its complexity and processing power, ranging from millions to billions of parameters, which is why they are called “large” [17]. LLMs use advanced deep learning (DL) architectures to independently learn intricate linguistic patterns and semantics from extensive training data, setting them apart from approaches that depend heavily on predefined rules and feature engineering. This allows LLMs to exhibit human-like abilities in understanding and producing text, summarizing information, and interpreting contextual cues with impressive accuracy [3].

LLMs have a broad range of applications in medicine, including drug discovery, clinical decision support, patient care, research, documentation, medical education, and licensing [18]. They significantly enhance diagnostic accuracy, streamline downstream processes, and improve patient outcomes [19]. Additionally, LLMs can continuously learn from new medical knowledge, making them adaptable and relevant in changing clinical environments, and supporting ongoing development in medical practice. These models serve diverse users, including clinicians, researchers, educators, students, and patients. Their use spans administrative tasks, documentation, clinical decision-making, educational tools, patient communication, and clinical NLP [20,21].

### 2.2. Study Design and Participants

This cross-sectional comparative study was conducted between 20 June 2024 and 2 December 2024 (Clinical trial number: not applicable). We compared the responses of seven LLMs and five early-career interventional cardiologists to 12 simulated Cath lab emergencies.

The five comparator physicians were defined as early-career interventional cardiologists with less than two years of independent practice, consistent with SCAI definitions [22,23]. The 30 evaluators were experienced operators, each with at least 5 years of independent professional experience. Although the number of scenarios was limited to twelve, this sample size aligns with prior LLM evaluation studies in digital health research, such as Neo et al. [24] and Anaya et al. [25], which used 10–12 question-based clinical assessments to evaluate chatbot performance.

The study’s Internal Review Board (IRB) was approved by Northwestern University under number STU00218531, Approval Date: 23 January 2023. All participants provided informed consent before being included in the study. Written consent was obtained from all physician participants and evaluators involved in the simulation and scoring process. No patient data were used; therefore, patient consent was not necessary.

### 2.3. Physician Comparator Group and Scope of Assessment

The comparator group of physicians consisted solely of early-career interventional cardiologists. Therefore, the scores they received reflect early-career cognitive abilities and should not be generalized to expert or attending interventional cardiologists. There could be notable variability among individuals within this group, highlighting its heterogeneity. Furthermore, the study assessed only cognitive clinical reasoning based on text responses to simulated scenarios. It did not measure real-time procedural skills, angiographic image interpretation, electrocardiographic waveforms, hemodynamic data, or responses under time constraints. Consequently, these results do not directly reflect real-world catheterization laboratory performance and should be considered within the scope of a simulated, reasoning-centered assessment.

### 2.4. Clinical Scenarios

Twelve challenging clinical scenarios were developed by a senior interventional cardiologist. These scenarios simulated potential intra-procedural complications and decision points during the management of a 55-year-old patient presenting with inferior myocardial infarction (MI) (Supplementary File).

### 2.5. LLM Prompting and Response Processing

The scenarios were posed to seven LLMs.

- ChatGPT (OpenAI, GPT-4o, version released in 2024);
- Claude (Anthropic, Claude 3 Opus);
- Gemini (Google, Gemini Advanced/Ultra 1.0);
- Llama (Meta, Llama 3 70B);
- Qwen (Alibaba, Qwen 2 72B);
- Bing Copilot (Microsoft; GPT-4–class large language models);
- Deep Seek (DeepSeek-V2).

**Figure 1:**
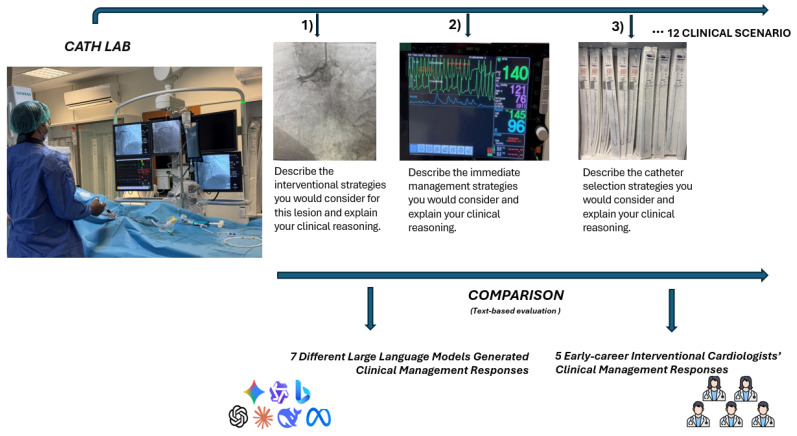
Study workflow illustrating standardized simulated catheterization laboratory scenarios presented to large language models and early-career interventional cardiologists, with anonymized, randomized responses evaluated under blinded conditions by expert reviewers.

All models were accessed via their respective web interfaces in July 2024. A standardized zero-shot Chain-of-Thought prompt structure was used, beginning with the persona assignment: “I am an interventional cardiologist.” The questions were first posed to seven LLMs, including OpenAI ChatGPT (GPT-4o, accessed via web interface), Google Gemini (Gemini Advanced/Ultra 1.0, web interface), Anthropic Claude (Claude 3 Opus, web interface), Meta Llama (Llama 3 70B, Application Programming Interface (API)), Alibaba Qwen (Qwen 2, API), Microsoft Bing Copilot (GPT-4-Turbo, web interface), and Deep Seek (DeepSeek-V2, web interface). Figure 1 illustrates a schematic overview of the study comparing seven LLMs with five early-career interventional cardiologists across 12 simulated Cath lab emergency scenarios. A standard prompt was applied across all scenarios to eliminate instructional framing bias; the underlying clinical content, scenario complexity, and evaluation methodology remained unchanged. LLM responses were generated using structured prompts that elicited explicit stepwise reasoning. Human participants were not provided with equivalent cognitive scaffolding, which may introduce an asymmetry favoring LLM outputs. This design choice reflects common benchmarking practice but constitutes a methodological limitation when comparing human and model performance. Standardization of LLM responses was limited to removal of formatting, disclaimers, and conversational elements; however, the possibility that subtle aspects of clinical emphasis or tone were affected cannot be fully excluded.

### 2.6. Response Standardization

To ensure fair comparison and effective blinding, LLM-generated responses underwent a standardization process (referred to in the original manuscript as “standardizations”). An interventional cardiologist reviewed the LLM outputs and applied standardized formatting (e.g., removing disclaimers or conversational filler) to match the style of the physician responses. Crucially, this process strictly preserved the original clinical content and management suggestions generated by the LLMs; no clinical information was added, deleted, or corrected.

### 2.7. Evaluation and Blinding

The standardized responses from the seven LLMs and five physicians were compiled into an examination paper, with responses displayed side-by-side in randomized order (Supplementary File).

To ensure blinding, the study design was presented to the 30 evaluators as follows: “This study aims to compare the knowledge of twelve fellows with varying lengths of experience in catheterization laboratory rotations.” The involvement of LLMs was not disclosed. This design ensured methodological rigor comparable to controlled experiments in digital health evaluation frameworks.

Evaluators scored each response on a scale from 0 (worst/unsafe) to 10 (best/optimal management). The grading rubric emphasized clinical appropriateness, safety, adherence to guidelines, and completeness of the management strategy.

### 2.8. Prompting Strategy

All interactions with the LLMs followed a standardized zero-shot persona-based Chain-of-Thought prompting framework to ensure consistency and minimize bias. In zero-shot prompting, the model is given a task, such as managing a patient with myocardial infarction, without prior examples, relying solely on its pre-trained knowledge and reasoning skills. This approach was chosen to emulate real-world situations where clinicians ask AI systems questions without providing specific examples [26,27]. In contrast, few-shot prompting involves supplying the model with a few question–answer pairs before testing, which helps it identify task patterns. However, this was intentionally avoided to prevent information sharing across cases and to maintain scenario independence [28].

To improve logical transparency, we added Chain-of-Thought prompting, which explicitly instructs the model to “think step by step” or “explain the reasoning process before providing the final answer.” [29,30]. Previous research has shown that Chain-of-Thought prompting encourages models to generate intermediate reasoning steps that resemble human analytical thinking, thereby enhancing interpretability and factual accuracy in high-stakes decision-making tasks [31].For full transparency and reproducibility, the complete standardized prompt text used across all scenarios is provided in the Supplementary Materials.

Each LLM received the same structured instruction block consisting of three parts (Supplementary File):

1. Persona declaration (Persona-based prompting): The prompt begins with “I am an interventional cardiologist,” establishing the expert identity. This prompts the model to reason using specialized interventional terminology, procedural priorities, and complication-management strategies.
2. Clinical scenario with no prior examples (zero-shot prompting): The prompt does not include any example cases or pre-defined model outputs.
3. Stepwise or task force structured reasoning (Chain-of-Thought prompting): Prompts were designed to elicit stepwise clinical reasoning without directive or hierarchy-implying language.

### 2.9. Statistical Analysis

The mean score and corresponding 95% confidence interval (CI) were estimated for each model, assuming normality. A combined violin–spaghetti plot was generated to visualize the distributions of inter-reviewer scores for both LLMs and doctors. To estimate the overall mean score and 95% CI across the five doctors, a linear mixed-effects (LME) model was employed to account for the correlation among scores from the same reviewers. The LME framework was selected because each of the 30 reviewers evaluated all 12 entities (seven LLMs and five physicians), leading to repeated-measures and non-independence of reviewer scores. This model appropriately accounts for within-reviewer correlation and allows simultaneous estimation of fixed effects (model type) and random effects (reviewer variability). Dunnett’s method was applied to adjust for multiple comparisons between each LLM and the physicians. All analyses were performed using R version 4.4.1, and statistical significance was determined at *p* < 0.05.

## 3. Results

A total of 12 clinical scenarios depicting potential challenges in managing a patient with inferior myocardial infarction in the catheterization lab were presented to 7 LLMs and 5 physicians. Their responses were standardized and compiled into an exam-style format (Supplementary File). Thirty independent interventional cardiologists, each with over five years of experience, assessed these responses using a 0–10 grading scale. Evaluators were instructed to score each answer based on predetermined criteria emphasizing clinical safety, diagnostic and therapeutic accuracy, adherence to current, structured, and completeness of management reasoning. A score of 0 indicated unsafe or inappropriate management, while a score of 10 represented an optimal, evidence-based, and comprehensive response.

### 3.1. Group Performance

In the overall evaluation (maximum total score: 120 points), the five attending cardiologists achieved an average score of 80.68 (95% CI [76.3, 85.0]). ChatGPT achieved the highest mean score among the evaluated LLMs 87.4 (95% CI [82.5, 92.3]), and showed numerically higher scores than the early-career physician reference group. Claude and DeepSeek achieved mean scores of 80.8 (95% CI [75.7, 85.9]) and 78.7 (95% CI [72.9, 84.6]), respectively, indicating performance comparable to that of human experts. LLAMA achieved a moderate score of 73.7 (95% CI [67.2, 80.2]), while Qwen and Bing performed lower with scores of 66.2 (95% CI [59.6, 72.9]) and 64.3 (95% CI [58.0, 70.6]), respectively. Gemini performed the worst among the models, with a mean score of 59.0 (95% CI [52.8, 65.2]), well below both physicians’ and other LLMs’ (Table 1).

**Figure 2:**
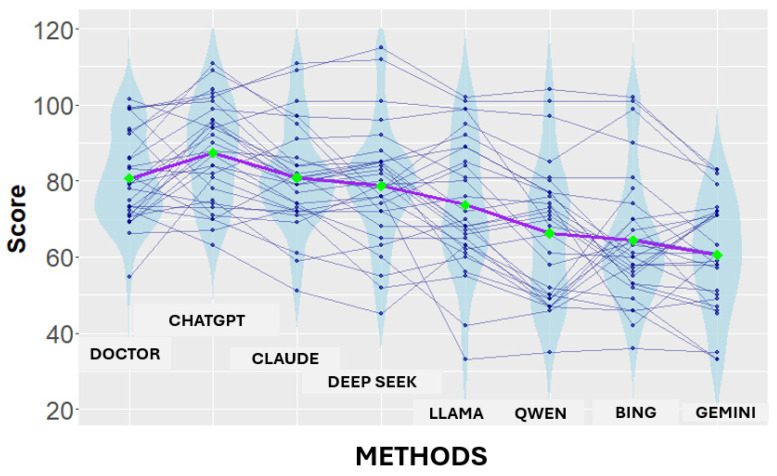
Violin–spaghetti plot showing the distribution of reviewer-assigned scores for large language models and physician comparators, reflecting subjective expert assessment of simulated clinical reasoning rather than objective clinical outcomes. Colored violins depict the score distribution for each model and physician group, while thin color-matched spaghetti lines represent individual reviewer score trajectories across groups, illustrating inter-reviewer variability and consistency.

To better visualize these results, a combined violin–spaghetti plot was created (Figure 2). Each violin shows the distribution of reviewer-assigned scores for a specific model, while individual spaghetti lines connect each reviewer’s scores across all models, illustrating intra-reviewer variability. Green diamonds indicate mean scores, and the order of models from left to right is based on these mean values. This visualization emphasizes both the consistency and dispersion of reviewer assessments and clearly shows ChatGPT’s numerically higher and stable performance compared to other models and physicians. These findings are not only statistically significant but also have major implications for AI governance, showing how publicly accessible models can differ greatly in safety-critical aspects contexts.

The key finding is the emergence of a high-performing subset of LLMs: ChatGPT’s mean score was statistically higher than that of the early-career physician reference group (*p* < 0.05), while Claude and Deep Seek achieved similar accuracy levels. In contrast, Gemini, Qwen, Bing, and LLAMA showed lower and more inconsistent scores, highlighting the variability in reliability and contextual reasoning among current-generation models.

### 3.2. Statistical Comparisons

Linear mixed-effects modeling was used to compare each LLM with physicians’ performance. ChatGPT scored marginally higher than physicians, with a mean difference of 6.69 (95% CI [0.00, 13.37]; *p* < 0.05). Claude and Deep Seek showed no significant differences compared with physicians (0.15 [−6.71, 7.02]; *p* = 1.000 and −1.95 [−8.94, 5.04]; *p* = 0.906, respectively). By contrast, Gemini, LLAMA, Qwen, and Bing all scored significantly lower than physicians, with mean differences ranging from −6.98 (95% CI [−13.46, −0.29]; *p* = 0.036) to −21.65 (95% CI [−27.93, −15.37]; *p* < 0.001) Table 2.

### 3.3. Interpretation

These findings underscore the variability among LLMs in handling emergency interventional cardiology cases. While ChatGPT demonstrated a higher mean score than the early-career physician reference group, Claude and DeepSeek showed similar performance, indicating potential clinical usefulness. In contrast, Gemini consistently performed worse, and LLAMA, Qwen, and Bing achieved weaker results than the physician reference group.

## 4. Discussion

Our comprehensive analysis highlights the potential of LLMs as valuable decision-support tools in emergency cardiac interventional settings, while also revealing significant variability in their clinical reasoning performance. Among the seven LLMs tested, ChatGPT demonstrated the highest mean scores among the evaluated LLMs and performance comparable to that of early-career interventional cardiologists in this simulated setting. Claude and Deep Seek achieved results statistically comparable to those of doctors, suggesting that certain advanced LLMs can approach expert-level decision-making when appropriately prompted. In contrast, Gemini, LLAMA, Qwen, and Bing showed markedly lower scores, often producing fragmented or overly generalized responses that lacked procedural prioritization or safety considerations. Overall, these findings indicate that certain LLMs could act as supportive cognitive tools for early-career interventional cardiologists by offering structured, guideline-aware clinical reasoning during complex catheterization procedures. Notably, ChatGPT demonstrated numerically higher mean scores than the early-career physician reference group in this simulated setting. The variability in performance among LLMs emphasizes key factors for future AI benchmarking and safety assessments. These results highlight that LLMs are not interchangeable, and their specific reliability must be thoroughly evaluated before considering them for clinical decision-support roles. Notably, these findings are based on simulated, text-based scenarios and should be viewed as exploratory rather than reflective of the impact of actual clinical or population-level settings [32]. This study extends prior evaluations of LLMs in high-risk clinical reasoning by examining simulated catheterization laboratory emergency scenarios using blinded expert assessment. These findings should be interpreted as exploratory and hypothesis-generating, reflecting comparability within a limited early-career physician benchmark rather than evidence of clinical superiority or equivalence to experienced interventional cardiologists. Our findings should be interpreted in the context of prior high-risk AI studies in acute care, which have primarily evaluated diagnostic or triage-oriented tasks; the present work extends this literature to simulated procedural decision-making in the catheterization laboratory.

The wide performance range of LLMs highlights the diversity in model training architectures, data sources, and reasoning optimization strategies within current LLM ecosystems [33]. Models primarily trained on broad, non-specialized internet corpora often display high linguistic fluency but have limited domain-specific procedural reasoning and inconsistent adherence to established clinical guidelines [34]. Conversely, models that incorporate reinforcement learning, supervised instruction tuning, and exposure to curated medical datasets tend to achieve higher contextual accuracy, greater clinical relevance, and better alignment with safety standards. This variability shows that LLM performance strongly depends on the specific model rather than being interchangeable, and its reliability in real-world clinical decision support largely relies on its underlying design, domain adaptation, and data sources [35,36]. Among 7 LLMs, ChatGPT demonstrated the best performance in Cath lab challenges.

LLMs have become ideal tools for transforming human–machine interactions in healthcare due to their ability to generate coherent, contextually appropriate text [37]. In cardiology, LLMs can support medical diagnosis and decision-making by integrating patient symptoms, medical history, and other relevant data to enhance diagnostic accuracy and inform treatment plans [38,39,40]. Beyond this, AI systems can analyze diagnostic images, improving the accuracy of assessments for cardiology patients. LLMs also customize patient education resources, converting complex medical information into accessible content for diverse audiences, making them a vital part of patient-centered care. [40]. The use of LLMs in cardiology is rapidly growing, especially for detecting abnormalities such as arrhythmias and ST-segment changes, combining data with other diagnostic methods, and forecasting cardiac disease risk [41]. In a study by Zhu et al. [42], it was shown that GPT-4 achieved an accuracy rate of 83.87% in multiple-choice ECG questions in various medical exams. AI and language models can support the diagnosis and management of various cardiovascular diseases. Li et al. [43] Demonstrated that LLMs answered 25 questions in the complex medical field of cardio-oncology with an accuracy rate of 68%. In our study, LLMs, especially ChatGPT, performed slightly better than early-career doctors, while Claude and Deep Seek delivered results comparable to those of doctors in patient management within the Cath lab.

LLMs are based on the Generative Pre-trained Transformer (GPT) architecture. These models are trained on large datasets and can engage in dynamic conversations across multiple languages [44]. Through plugin integrations, LLMs can access real-time information and knowledge-based data; they can also process both textual and visual inputs [44]. In our study, ChatGPT alone performed significantly better than the early-career cardiologist group, consistent with findings from a recent study using a different LLM technology. This difference is likely due to the sensitivity of LLM outputs to prompt design and formatting [45]. There are many conceptual frameworks for prompt construction that aim to guide LLM behavior. Most of these approaches focus on clearly defining the task, context, and instructions; our prompts were developed iteratively in line with these established frameworks [46,47].

ChatGPT’s strong performance likely results from a balance between thoroughness and focused reasoning, rather than mere verbosity. As in educational LLM research, models like GPT-4 tend to achieve higher accuracy when guided by structured, context-aware reasoning methods such as chain-of-thought (CoT) prompting [48]. In our study, ChatGPT not only provided more detailed responses but also emphasized domain-specific information, highlighting immediate life-saving actions while explaining the underlying pathophysiology. This aligns with the results from Lee et al. [49], where few-shot and CoT-guided GPT-4 outputs achieved greater accuracy and interpretability by following explicit reasoning steps. Therefore, ChatGPT’s higher scores probably stem from its ability to synthesize concise, clinically relevant decisions within a clear explanatory framework that focuses on the correct priorities rather than exhaustive lists. This structured reasoning, rather than response length, helped ChatGPT better mimic the decision-making patterns of expert cardiologists in acute myocardial infarction cases.

The potential utility of LLMs in the Cath lab is multifaceted. For early-career cardiologists, who often face high-pressure decision-making [4], tools like ChatGPT could provide real-time, expert-aligned insights, potentially reducing cognitive load and enhancing procedural safety. By offering immediate access to synthesized knowledge, LLMs may help standardize care during critical procedures. It is crucial to emphasize that these technologies are emerging as complementary tools rather than replacements for human expertise [40]. Any potential clinical uses of LLMs should remain strictly supportive tools under clinician supervision. Their outputs should serve as decision-support aids that summarize options, provide a reminder of differential steps, or highlight guideline-related factors, rather than as definitive recommendations. This is crucial because LLM performance can vary significantly depending on how prompts are framed, and in critical situations, they might confidently provide incorrect or incomplete advice. In settings like the catheterization lab, ensuring safety would involve safeguards such as human oversight, validation against real-time clinical data, transparent communication about uncertainty, and clear accountability structures, aligned with international ethical guidelines for AI in healthcare [50].

Despite their promise, the deployment of LLMs in medicine faces significant challenges [51]. Concerns regarding accuracy and the potential for generating plausible but incorrect information (“hallucinations”) remain paramount. Furthermore, LLMs are known to exhibit cognitive biases derived from their training data. Studies indicate that LLMs may display stronger cognitive biases than clinicians in certain contexts, which can influence treatment options [52]. Recognizing, measuring, and mitigating these biases is essential for the safe and ethical integration of LLMs into the Cath lab environment.

This study has several limitations. First, the simulated scenarios, while complex, cannot fully replicate the dynamic nature of real-world medical emergencies, nor do they incorporate multimodal data (e.g., angiograms or hemodynamic waveforms). Second, the physician comparator group was limited in size and consisted exclusively of early-career interventional cardiologists. In addition, performance was evaluated using expert subjective scoring of simulated scenarios rather than safety-weighted or outcome-based metrics, which limits the generalizability and clinical interpretability of comparative claims. Third, the study focused exclusively on inferior MI scenarios; generalizability to other cardiovascular emergencies requires further investigation. Fourth, the physician benchmark group comprised early-career cardiologists comparisons with senior experts might yield different results. Fifth, although evaluators were blinded to the involvement of LLMs and were informed that fellows with varying levels of experience generated all responses, complete blinding cannot be guaranteed. Despite content-preserving standardization, stylistic features characteristic of AI-generated text may have been detectable to experienced evaluators, potentially influencing scoring. No formal sensitivity analysis was performed to assess the impact of suspected AI detection on evaluator behavior, and neither favorable nor unfavorable subconscious bias can be excluded. This limitation is inherent to comparative evaluations of human and AI-generated free-text responses and should be considered when interpreting performance differences. Finally, the study design includes potential sources of bias favoring LLMs, including structured reasoning prompts and post hoc response standardization. Although no clinical information was intentionally added or removed, subtle influences on prioritization or risk emphasis cannot be definitively ruled out. These factors limit the interpretability of direct performance comparisons. Future studies should assess the generalizability of these findings across other myocardial infarction phenotypes (e.g., anterior STEMI, NSTEMI) and additional cardiovascular emergencies. Benchmarking LLM performance against senior or expert interventional cardiologists will be essential to contextualize results relative to higher levels of clinical experience. Moreover, because real-world catheterization laboratory decision-making is inherently multimodal, future evaluations should incorporate angiographic images, electrocardiographic waveforms, invasive hemodynamic data, and time-pressure constraints. Prospective, multicenter studies integrating these elements will be necessary to determine the reliability and safety of LLM-assisted clinical reasoning in procedural cardiology.

Our research provides a rigorous benchmark for LLM performance in emergency interventional cardiology scenarios. ChatGPT, Claude, and Deep Seek demonstrated a remarkable ability to generate clinically sophisticated responses, performing comparably to early-career cardiologists in this simulated setting. Although significant variability exists among current LLMs, the findings suggest a future in which high-performing models can serve as intelligent assistants in high-stakes medical settings, supporting healthcare providers in rapid, contextually relevant decision-making. These findings should be interpreted as exploratory and hypothesis-generating rather than as evidence of clinical superiority or real-world safety.

## Figures and Tables

**Table 1:** Mean performance scores of large language models (LLMs) and physicians (DR.1–DR.5) in emergency cardiac catheterization scenarios.

Model/Physician	Mean ± SD	SE	95% CI (Lower–Upper)
ChatGPT	87.4 ± 13.0	2.40	82.5–92.3
Claude	80.8 ± 13.6	2.49	75.7–85.9
Deep Seek	78.7 ± 15.7	2.87	72.9–84.6
LLAMA	73.7 ± 17.4	3.17	67.2–80.2
Qwen	66.2 ± 17.9	3.26	59.6–72.9
Bing	64.3 ± 16.8	3.07	58.0–70.6
Gemini	59.0 ± 16.6	3.02	52.8–65.2
DR.1	78.9 ± 16.7	3.05	72.6–85.1
DR.2	68.4 ± 16.7	3.04	62.1–74.6
DR.3	81.0 ± 15.0	2.73	75.4–86.6
DR.4	76.7 ± 16.3	2.97	70.6–82.7
DR.5	98.5 ± 10.8	1.97	94.4–102.0

Mean for five cardiologists: 80.68 (95% CI [76.3, 85.0]).

**Table 2:** Comparative performance of large language models (LLMs) versus physicians based on linear mixed effects models.

Comparison	Difference in Mean Score	95% CI (Lower–Upper)	*p*-Value
ChatGPT vs. physicians	6.69	0.01–13.36	<0.050
Claude vs. physicians	0.15	−6.71–7.02	1.000
Deep Seek vs. physicians	−1.95	−8.94–5.04	0.906
LLAMA vs. physicians	−6.98	−13.46–−0.50	0.036
Qwen vs. physicians	−14.45	−20.52–−8.37	<0.001
Bing vs. physicians	−16.35	−22.49–−10.20	<0.001
Gemini vs. physicians	−21.65	−27.93–−15.37	<0.001

## Data Availability

The original contributions presented in this study are included in the article/Supplementary Material. Further inquiries can be directed to the corresponding author.

## Supplementary Materials

The following supporting information can be downloaded at: https://www.mdpi.com/article/10.3390/medsci14010033/s1, Table S1: Structure of the exam paper of 12 emergency clinical scenarios.
