# Supplementary material for: AI in the Hot Seat: Head-to-Head Comparison of Large Language Models and Cardiologists in Emergency Scenarios
Source: Med Sci (Basel). 2026 Jan 8;14(1):33. doi: 10.3390/medsci14010033 (PMC12821637; doi:10.3390/medsci14010033)
Supplement: Supplementary file 1 [file medsci-14-00033-s001.zip › medsci-4061459-supplementary.pdf]

**1**. I am an interventional cardiologist. I planned a percutaneous coronary intervention procedure for a 55-year-old patient due to inferior myocardial infarction. However, the patient's pain does not go away despite morphine and the patient is very active and I cannot continue the procedure. What do you recommend in this case?
**2**. I am an interventional cardiologist. I planned percutaneous coronary intervention for a 55-year-old patient due to inferior myocardial infarction. However, the patient's blood pressure is low and VT attacks are frequent, and the patient loses consciousness. In this case, what medicines should I give to the patient first?
**3.**I am an interventional cardiologist. I am considering performing a percutaneous coronary intervention on a 55-year-old patient who I suspect has an inferior myocardial infarction based on his ECG. I cannot engage the patient's RCA artery with Judkins catheter reason for its anterior superior exit. Which alternative catheter do you recommend?
**4.**I am an interventional cardiologist. I am considering performing percutaneous coronary intervention on a 55-year-old patient who I suspect has an inferior myocardial infarction based on his ECG. I engaged the patient's RCA artery with a no side hole Judkins catheter. The RCA proximal spasmed, it was completely blocked, and the patient's blood pressure dropped. What should I do first in this situation?
**5.**I am an interventional cardiologist. I planned a percutaneous coronary intervention procedure for a 55-year-old patient due to inferior myocardial infarction. RCA imaging shows widespread thrombus from proximal to distal. What would be my first choice when planning an intervention for this lesion?
**6.** I am an interventional cardiologist. I will place a stent in the PD branch of the RCA artery of a 55-year-old patient for whom I performed a percutaneous coronary procedure due to inferior myocardial infarction. However, cannot advance the stent from the proximal RCA reason for very calcified and tortuous. What would you suggest in this situation?
**7.** I am an interventional cardiologist. I planned a percutaneous coronary intervention procedure for a 55-year-old patient due to inferior myocardial infarction. There is a lesion in the RCA PD branch. But the proximal part of the RCA is very calcified and tortuous. While taking the stent to the lesion area, the stent peeled off and remained in the proximal RCA. In this case, how can I remove that stent from there?
**8.** I am an interventional cardiologist. No-reflow developed after opening the RCA of a 55-year-old patient on whom I had performed percutaneous coronary intervention due to an inferior myocardial infarction. In this case, what is the first-choice drug I should use, and can you tell me its dosage and method of administration?
**9.** I am an interventional cardiologist. I placed a stent in the RCA artery of a 55-year-old patient for whom I performed a percutaneous coronary procedure due to inferior myocardial infarction according to his ECG. Full patency was achieved. The patient's pain is gone. But the hazy appearance inside the stent continues. What would you recommend in this case?
**10.** I am an interventional cardiologist. I performed percutaneous coronary intervention on a 55-year-old patient due to inferior myocardial infarction. I chose the right radial artery as the access route and placed a stent in the PD branch of the patient's RCA artery, flow was established, and I completed the procedure. However, since the procedure took a long time, radial artery spasm occurred and I cannot withdraw my catheter. What should I do first in this case?
**11**. I am an interventional cardiologist. I planned percutaneous coronary intervention for a 55-year-old patient due to inferior myocardial infarction. I determined that the patient's RCA lesion was due to spontaneous coronary artery dissection. Considering the patient's left system angiography and echocardiographic examination, I think that the RCA feeds a very large area. How should I proceed with the procedure in this case?
**12**.I am an interventional cardiologist. I planned a percutaneous coronary intervention procedure for a 55-year-old patient due to inferior myocardial infarction. However, the patient has acute renal failure and I want to take a maximum of 4 exposures for the first imaging. In this case, which 4 exposures would be the most optimal in coronary angiography?

Table S1: Structure of the exam paper of 12 emergency clinical scenarios.
